# Resolving authorship disputes by mediation and arbitration

**DOI:** 10.1186/s41073-018-0057-z

**Published:** 2018-11-16

**Authors:** Zen Faulkes

**Affiliations:** Department of Biology, The University of Texas Rio Grande Valley, 1201 West University Drive, Edinburg, TX 78539 USA

**Keywords:** Authorship, Alternative dispute resolution

## Abstract

**Background:**

Disputes over authorship are increasing. This paper examines the options that researchers have in resolving authorship disputes. Discussions about authorship disputes often address how to prevent disputes but rarely address how to resolve them. Both individuals and larger research communities are harmed by the limited options for dispute resolution.

**Main body:**

When authorship disputes arise after publication, most existing guidelines recommend that the authors work out the disputes between themselves. But this is unlikely to occur, because there are often large power differentials between team members, and institutions (e.g., universities, funding agencies) are unlikely to have authority over all team members. Other collaborative disciplines that deal with issues of collaborative creator credit could provide models for scientific authorship. Arbitration or mediation could provide solutions to authorship disputes where few presently exist. Because authors recognize journals’ authority to make decisions about manuscripts submitted to the journal, journals are well placed to facilitate alternative dispute resolution processes.

**Conclusion:**

Rather than viewing authorship disputes as rare events that must be handled on a case by case basis, researchers and journals should view the potential for disputes as predictable, preventable, and soluble. Independent bodies that can offer alternative dispute resolution services to scientific collaborators and/or journals could quickly help research communities, particularly their most vulnerable members.

## Background

Academic authorship is an example of attribution [1], where people’s career reputation is based on credit for work they have performed. Many problems surrounding attribution stem from one of two issues: when contributors cannot predict how credit will be given [1], and when attribution is dictated by individuals with power [1]. Both conditions are common in some research fields of academic publishing, contributing to the likelihood of authorship disputes.

There are no widely accepted criteria for what constitutes authorship [2–4]. Individual journals do not always provide authorship guidelines [5, 6]. Researchers may work under multiple authorship guidelines (e.g., funding agency, institution [7], journal) that could conflict with each other. Currently, the closest guidelines that approach a widely accepted standard are recommendations for paper authorship in biomedicine from the International Committee of Medical Journal Editors [8], also known as the Vancouver guidelines. But even in biomedical research, the field for which they were developed, some journals do not describe the guidelines correctly [5], and many authors are ignorant of these guidelines [9–11] and/or disagree with them [10–12]. Because the Vancouver guidelines are rarely followed by journals that purport to abide by them [13–15], and were created from the top down by journal editors, these guidelines are examples of both “arbitrariness” and “power differential” factors that contribute to attribution failures [1].

The Vancouver guidelines [8] only try to answer, “Who should be an author?” They provide no guidance for other contentious points regarding authorship, which increases the possibility of authorship being assigned arbitrarily. For multi-author papers, there are at least three designations that are often viewed as more important than others: first author, last author (also sometime called senior author), and corresponding author [6, 7, 16–18], of which the last is the only author designation that the Vancouver guidelines address [8]. The first author is generally assumed to be a person (often an early career researcher) who has done the largest portion of the experimental work and the writing, and who deserves most of the credit. The last author is generally assumed to be an individual (often in a more advanced career stage) who is providing overarching intellectual questions, funding, and writing, but has not necessarily been directly involved in data collection. Some research projects use last authorship as a proxy measure for career stage [19, 20]. The author of one study described last authorship as, “the pinnacle of the research career and has a lot of status that goes along with it” [21]. Authors between first and last are generally expected to have made smaller contributions, listed in order of effort (i.e., second author contributed more than third, etc.) [6]. The expectations of corresponding authorship vary but the designation is often thought to indicate overall responsibility for the project [8, 18, 22]. Empirical research generally supports these interpretations [16], but in some fields, author order is alphabetical and contains no information about contribution.

These practices mean that academic authorship is not only a valuable resource; it can be a limited resource. That some authorship designations are more valuable to career advancement than others [19] increases the incentives for people to game authorship systems and creates more reasons for disputes. The number of authors on journal articles has been increasing in many fields [16, 22–27]. The current record-holder, in particle physics, is a paper with 5,154 authors [28], but biology papers have also cracked the 1,000 author mark [29]. As author lists have increased, the problems of ascertaining and assigning credit by authorship (or, if the paper is flawed, blame) [30] have increased, as have the number of disputes over authorship [31].

People have used creative ways to spread the benefit of receiving key authorship credits. An increasingly common practice is to use author’s notes to designate equal contributions [32–34]. The record for greatest number of “equally contributing” authors is unknown, but a cursory search of recent issues of journals quickly found a paper with seven authors (out of 44) listed as having made equal contributions, and none were first author [35]. One article with four authors designated that all contributed equally (creating the linguistic puzzle of whether they should be called “co-first” authors or “co-senior” authors), and listed all as corresponding authors [36], making all three designations effectively meaningless. Journals have generally not adopted policies or guidelines for equal contribution statements [33], nor are there general practices for handling such notes in research evaluations [34]. Contribution notes notwithstanding, the first author’s name becomes the most associated with the paper because many journals's citations in the body of the text list only the first author when there are three or more, and “et al.” sweeps away whatever information is conveyed by fine print about equal contribution [2, 37–42].

The combination of many people (e.g., large research teams) given vague guidance (e.g., authorship guidelines) about distributing limited resources (e.g., first authorships) with clear career consequences (e.g., hiring and promotion) make authorship disputes a completely predictable outcome. Studies on the prevalence of authorship disputes usually report somewhere between a third to two-thirds of researchers have been involved in authorship disagreements [10, 43–46], but some of those may have been resolved before publication. It is difficult to know how many projects have never been published because of unresolved authorship disputes, although it is not zero [41]. Researchers not submitting papers because of disputes would explain why journal editors do not think authorship disputes are a severe problem [47], with 29% of editors reporting authorship disputes never happen at their journal [47].

Many papers discuss the importance of dispute prevention, often mentioning the need for clear guidelines and extensive communication about authorship expectations between collaborators, mentors, and journals [5, 12, 31, 32, 37, 48–52]. But it is not reasonable to expect these policies to prevent all problems [53]. The existence of business contracts, for example, does not remove the possibility of lawsuits over failing to meet contractual obligations. There is less discussion of dispute resolution (but see [54]), which is the focus of the remainder of this paper, particularly disputes that are not resolved when a paper is published. Papers are routinely retracted because of authorship disputes [55], with one study showing authorship disputes contributed to 7.4% of retractions [56]. Reasons for retraction include author omission [57, 58] and disagreements over author placement [59]. Because retractions represent serious sanctions and are rare (but increasing) [56, 60–63], retractions probably record only a fraction of authorship disputes. There are many unanswered questions about how authorship disputes affect research publications and career advancement.

## Authorship disputes are difficult to resolve

Disputes can harm both the individuals involved and the communities they belong to. Communities usually have practices intended to prevent disputes before they occur and resolve them if they do occur. Good policies and guidelines can help prevent disputes or help parties involved resolve their own fights. Even with good policies, however, disputes can still happen. People expect communities to have mechanisms to help resolve disputes (e.g., policing, legal action, counseling). If two neighbors fight, others in the community may hope they sort it out themselves, and combatants might argue that only they have the right to end the fight. But it would be unethical for no community member to ever get involved in the fight, instead allowing the conflict to escalate until one person was dead. Having no way of resolving disputes in progress has corrosive effects on communities, particularly its most vulnerable members. Yet this is close to the reality of the situation in research communities for authorship disputes: researchers may struggle to find any help in resolving disputes.

The Committee on Publication Ethics [37] recommends that authorship disputes be resolved by the authors working out their differences on their own. There are many problems with trying to resolve such disputes internally. Power differentials contribute to attribution problems [1], and there are huge power differential between trainees and senior scientists. Senior faculty are most likely to be bullies [64, 65], making the potential for senior researchers to dictate authorship credit ripe for abuse [7, 65]. Some argue that nobody besides those involved in a project should ever be involved in an authorship decision because it would curtail academic freedom [7]. But this position favors the powerful and disenfranchises the vulnerable. Some evidence suggests that people who belong to underrepresented groups are more likely to be caught in disputes [31], and it seems likely such individuals are less likely to have disputes resolved to their satisfaction. Communities can and do limit the freedom of individuals to resolve their own disputes if the level of potential harm to participants is great enough, and loss of credit due to authorship disputes may be a little recognized factor driving underrepresented individuals out of scientific careers.

There is no guarantee that internal discussion will resolve the problem, despite clear incentives to do so, because journals reject or retract papers with authorship disputes [7, 66]. Retracting a paper because of an authorship dispute is a “scorched earth” solution where nobody wins. None of the authors win, because nobody gains credit for a retracted paper. Nor do readers win, since there was no implication that the science was unsound. Nor do funding agencies win, since their investment in the research sees no returns in publications.

If internal discussion fails to resolve the issue, there are no generally recognized avenues for authors to seek help in resolving it. Some options discussed below might exist for some authors, but there are no norms in the research community for dispute resolution.

A trainee might inform an institutional administrator, like a department chair or college Dean, who oversees the principal investigator of the project. But when faculty from multiple departments or institutions are involved in collaborative projects, it may not be clear who is the relevant administrator to discuss the dispute with [6]. Administrators may have no authority to act even if they are willing to step into an authorship dispute. Similarly, ombuds offices [31] or committees [7] at institutions could conceivably play a role, but not every institution has such offices. Research compliance or research integrity offices [50] might be relevant if misconduct was involved, but authorship disputes can arise that involve no misconduct. Because the standards for authorship placement are vague, any administrator or office charged with ensuring compliance might reasonably ask what standards the researchers are supposed to be complying with.

Authors might ask journal editors to resolve authorship disputes. By submitting a manuscript to a journal, authors implicitly recognize the editor’s authority to decide what goes into a journal. Some suggest this is part of an editor’s responsibility [18], and some editors find that authors are responsive to requests to shorten author lists [50]. But it seems unfair and unwise to expect editors to resolve authorship disputes on their own [37]. Editors probably do not have the local knowledge [37, 67] or resources to investigate the facts of a dispute thoroughly. Resolving authorship disputes would be a significant expansion of an editor’s responsibilities: it should not be their job. Having editors investigate disputes or make recommendations for authorship would create opportunities for conflicts of interest [68]. This is not to say that the editors and staff of journals should have no role in dispute resolution, however, as will be discussed below.

## Creator credits in other fields

There are many collaborative fields where attribution is contentious [1] that might provide models for science. For example, many popular comic characters were created by teams of writers and artists, who were often denied any credit for years [69]. Batman was first drawn by artist Bob Kane, but writer Bill Finger wrote many stories that defined the character and never received credit until after his death [70–72]. Spider-Man was sometimes credited as the creation of writer Stan Lee, prompting pushback from artists Jack Kirby [73, 74], who said he created the character, and Steve Ditko [75, 76], who said he co-created the character (with Lee generally agreeing with Ditko [75, 76]). The question of who created these iconic pop culture characters is more than a point of debate for comic book historians. Like academic authorship, there are clear financial and career rewards associated with creator credits. These characters earn huge amounts of money from comics, licensing, film, and television, and creator credit can ensure artists receive some of it. The financial stakes involved has meant that creator credit has been the subject of lawsuits or other legal actions by writers, artists, or their estates [69, 77]. Such legal action is hardly unique to comics [78]. But using courts to resolve on authorship credit on scientific papers is rare [54, 79]. The law is often neutral on questions of authorship, which is exacerbated by the lack of clarity about professional practices and ambiguous damages from denied authorship credit. Lawsuits are costly and lengthy [54]. In one case where an authorship dispute did go to court [79], the ruling favored the plaintiff who claimed first authorship, but the manuscript was apparently never published. No paper matching the description can be found in databases of scientific publications. Like the old joke, “The surgery was a success, but the patient died,” the plaintiff won the case, but science lost.

Another collaborative field where there are routinely credit disputes is screenwriting for television and movies in the USA [1, 32]. There are similarities between screenwriting and academic writing. First, both movie scripts and scientific articles often pass through the hands of many writers. Thirty-five people were involved in writing in *The Flintsones* movie, but only three names appeared on screen when the film was released [80]. Second, in both movie scripts and scientific articles, credit is complex and cryptic to outsiders [81]. For example, the writing credits for the movie *Lethal Weapon 3* read, “Screenplay by Jeffrey Boam and Jeffrey Boam & Robert Mark Kamen. Story by Jeffrey Boam.” Details like why Boam’s name is listed twice in the “Screenplay” credit or why names are joined with “and” versus an ampersand are as baffling to people unfamiliar with screenwriting conventions as scholarly authorship is to people outside academic fields. Unlike some of the cases with comics, however, disputes over screen credits usually go to arbitration rather than court. Usually, the Writer’s Guild of America is the final arbiter [80, 81]. The Writer’s Guild of America has established rules for determining who gets credit [82], albeit with room for interpretation, like what “substantial” means.

## Alternative dispute resolution in academic publishing

Mediation, arbitration, and combinations of these (e.g., med-arb [83, 84]) are examples of alternative dispute resolution [68]. They are “alternative” in the sense they are not resolved in courts. Alternative dispute resolution could be a valuable means for resolving authorship disputes that bypasses litigation [54].

A major difference between screenwriting and science is that Hollywood screenwriters are part of a single unionized workforce [1], while scientists lack any such central authority to compel them to seek arbitration or mediation. In cases where all authors were at the same institution, an institutional committee, or research ethics consultants might provide mediation [7, 85]. But the only authority that would be relevant to all authors of a manuscript, regardless of institutional affiliation, is the journal’s editorial staff. Authors implicitly recognize this authority when they submit a manuscript. But as discussed above, implicit authority alone does not mean editors are well positioned to perform arbitration or mediation. Rather, arbitration or mediation should be conducted not by editors, but by independent agencies (e.g., committees, businesses, non-governmental organizations, research ethics consultants) that specialize in alternative dispute resolution. These organizations would be staffed by people of diverse backgrounds who are experienced with scientific publishing, investigation, and dispute resolution. These agencies might be operated by a publisher, a journal, an institution, or a scientific society, but be independent from the editorial team, similar to the ethics committees (e.g., [86]) or journalists working at scientific journals. Dispute resolution agencies could provide services to many journals, not just one. In this model, editors would facilitate a process of alternative dispute resolution, not conduct it.

The tacit recognition of editorial authority could be made explicit. For example, when a journal accepts a paper, the editors could require authors to sign a form agreeing that by having this paper published in this journal, they would submit to binding arbitration if a dispute arises. Many journals already have such processes in place for copyright transference, payment of page charges or open access fees, and so on. Mandated arbitration poses potential ethical problems [68], so journals may not want to make arbitration a requirement for publication. Instead, journals could recommend mediation or arbitration only if disputes arise. Authors voluntarily agreeing to arbitration or mediation does not threaten academic freedom [7]. If the authors did not agree to binding arbitration, or mediation fails, the authors would be free to try to resolve the problem internally within a set time or face an editor’s decision by fiat. The key point is that “work it out by yourselves” becomes one of several options for authors in a dispute, not the only option.

The simplest scenario is one in which a dispute arises after a paper has been submitted to a journal. Depending on the journal’s specific policy, journal staff would either recommend mediation or arbitration, or simply initiate the process by contacting the alternative dispute resolution agency. Because alternative dispute resolution processes come in many forms [83, 84], journals might differ in what dispute resolution process they prefer. For papers that have already been published, however, some form of resolution including arbitration might be more appropriate than mediation alone because an editor needs to make a decision about a paper’s version of record. The mediators or arbiters would investigate, applying the generally accepted practices of the field, which would be known to authors in advance. For example, in life sciences, it would be expected that the author who performed the most tasks would be first author, and the author with the greatest seniority would be last. While authors may overvalue their own work [87], people engaged in dispute resolution in any field are routinely tasked with making decisions where participants give contradictory information (e.g., judges and juries in court). A mediation or arbitration process might be similar in some ways to a peer review system. There may be multiple mediators or arbiters who investigate the claims and facts of the dispute, perhaps with some specifically assigned to act as advocates for the different individuals, like prosecution and defense attorneys in court. The alternative dispute resolution committee or agency would deliver recommendations to the journal’s editor-in-chief, who would implement the decision.

Alternative dispute resolution could be supported by funds from publishers and journals, as part of their commitment to ethical publishing practices [88]. Assistance in resolving disputes could become a mark of excellence as a service that high-quality journals are expected to offer, like enlisting and coordinating the efforts of peer reviewers, editing, typesetting, copyediting, and promotion of articles to media. Funding agencies also have incentives to support the costs of alternative dispute resolution. Authorship disputes diminishes the return on funders’ investments by preventing publication of research they funded. Funding agencies might make arbitration or mediation permissible expenses for grants, like how many funding agencies started including article processing charges as allowable expenses for the agency to support open access publication. Alternately, funding agencies might set aside some funds for dispute resolution as a contingency and provide them to researchers on a case-by-case basis.

The description above focuses on dispute resolution occurring after a manuscript has been submitted to a journal. But alternative dispute resolution agencies could also be involved in dispute prevention by providing services to authors directly. This would be similar to independent businesses that assist with writing and editing (often for authors writing in languages that they are not fluent in) [89], which are separate from the review, copyediting, and proofing services provided by journals. Making alternative dispute resolution available to authors through independent businesses may prevent disputes from occurring or resolve them before manuscripts are submitted to journals, which would prevent errata or retractions. Research proposals, particularly collaborations between individuals at different institutions, could require that principle investigators submit plans for dispute resolution [6, 90], analogous to requirements that proposals include plans for data management. These plans could include dispute resolution services.

One advantage of alternative dispute resolution systems is that they increase transparency by providing a clear pathway for dispute resolution. The increased presence of alternative dispute resolution on the publishing landscape may encourage improved record-keeping, because clear documentation of the project’s progress would be essential to having a decision in one’s favor. The more authorship disputes go through arbitration or mediation and are resolved through that process, the more likely that authors will become aware of the need to talk to each other about their expectations for authorship, much like how early fights by comic creators changed practices in that industry [91, 92].

The model for alternative dispute resolution for authorship suggested here is similar to research ethics consultation services [85, 93]. These consulting services provide advice on topics that are not covered by regulatory agencies like institutional review boards (IRBs) [93], potentially including dispute resolution [85]. One consulting service was funded jointly by research funding agencies and a host institution [94], providing an example of how such agencies might be supported.

Creating alternative dispute resolution processes within academia faces a common problem that individual interests are not always aligned with community interests. People who now have the seniority to try to determine or influence authorship credit could have that power reduced if alternative dispute resolution services were well known and readily available to authors. But because many academics have personally experienced authorship disputes, they might see the value for the research community for having new mechanisms for resolving disputes. Furthermore, because authorship disputes reduce scientific productivity, stakeholders who are concerned with maximizing research outcomes (e.g., funding agencies) have incentives to join community leaders on this issue in raising awareness and creating new policies.

## Conclusion

Authorship disputes can be anticipated and ameliorated. While more can be done to prevent authorship disputes, the lack of options for resolving authorship disputes that occur is a major ethical challenge that the research community needs to address. To borrow from the old saying, an ounce of prevention may be better than a pound of cure, but when it comes to authorship disputes, it seems that currently, there are only ounces of prevention and no cure in any quantity. Alternative dispute resolution that is facilitated by journals’ staff but not necessarily run by them could help address this problem.
